# Serum creatinine to cystatin C ratio is a prognostic indicator in esophageal squamous cell carcinoma receiving neoadjuvant immunochemotherapy

**DOI:** 10.3389/fimmu.2025.1645874

**Published:** 2025-09-17

**Authors:** Jifeng Feng, Liang Wang, Xun Yang, Qixun Chen

**Affiliations:** ^1^ Department of Thoracic Surgery, Zhejiang Cancer Hospital, Hangzhou Institute of Medicine (HIM), Chinese Academy of Sciences, Hangzhou, Zhejiang, China; ^2^ Key Laboratory Diagnosis and Treatment Technology on Thoracic Oncology, Zhejiang Cancer Hospital, Hangzhou, Zhejiang, China

**Keywords:** esophageal squamous cell carcinoma, creatinine to cystatin C ratio, neoadjuvant immunochemotherapy, disease-free survival, overall survival

## Abstract

**Background:**

This study aimed to explore the relationship between pretreatment serum creatinine to cystatin C ratio (CCR) and prognosis in patients with esophageal squamous cell carcinoma (ESCC) receiving neoadjuvant immunochemotherapy (NICT).

**Methods:**

Two hundred and two ESCC patients who received NICT were included in the current retrospective study. The restricted cubic spline displayed the non-linear relationship between prognosis and CCR. The association between overall survival (OS)/disease-free survival (DFS) and CCR was also investigated. Kaplan-Meier methods and Cox proportional hazard regression analyses were employed.

**Results:**

The connection between DFS/OS and CCR suggested that their interaction was non-linear. The restricted cubic spline (RCS) model identified 97.5 as the ideal cutoff point for CCR and divided it into 2 groups. Patients exhibiting low CCR demonstrated significantly worse 3-year DFS (48.5% vs. 74.3%, P<0.001) and OS (62.9% vs. 82.9%, P=0.001) compared to those with high CCR. The results indicated that CCR had prognostic significance for the ESCC cases stratified according to subgroup analyses. Compared to the low CCR group, subsequent multivariate analysis revealed that the high CCR group reduced the risk of recurrence by 56.0% (P=0.001) and the risk of death by 51.8% (P=0.013), respectively.

**Conclusion:**

The therapeutic efficacy of NICT for ESCC can be predicted by pretreatment CCR. Although the CCR may not have attained a very high quality, it still holds certain significance for clinical practice in ESCC patients undergoing NICT.

## Introduction

Ranked as one of the most prevalent digestive tract cancers globally, esophageal carcinoma (EC) continues to impose a substantial public health challenge (1). Geospatial analysis reveals dramatic variance in EC prevalence, demonstrating 20-fold incidence differentials between endemic zones and low-risk territories (2). Pathological stratification delineates two primary forms, with esophageal squamous cell carcinoma (ESCC) being the main subtype (3). While therapeutic innovations encompassing neoadjuvant chemotherapy (NCT) and neoadjuvant chemoradiotherapy (NCRT) have enhanced survival, overall prognosis persists at concerning levels (4, 5). The therapeutic landscape of EC has witnessed paradigm shifts through immune checkpoint inhibitors (ICIs), particularly for those with metastatic disease (6, 7). Furthermore, for those with locally advanced EC, based on an increasing amount of evidence, neoadjuvant immunochemotherapy (NICT) appears to be efficacious and safe (8–10). However, more and more additional clinical practice validation is required to demonstrate the clinical efficacy in EC regarding NICT.

Currently, the most widely utilized methods for predicting prognosis in a variety of cancer patients are tumor-specific variables, such as pathological stage and perineural and/or vascular invasion (11). The prognosis of cancer patients is also greatly influenced by individual patient factors, such as nutritional-inflammatory status and immunological response, which are reflected in the form of hematological indicators (12). In daily clinical assessment, serum creatinine (CRE) and serum cystatin C (CYC) constitute clinically essential indicators for evaluating renal filtration capacity (13). Originating predominantly from muscular catabolic processes, CRE demonstrates diminished plasma concentrations in cancer patients experiencing muscle depletion, especially in those with sarcopenic syndromes (14). As a ubiquitously expressed protease inhibitor, conversely, CYC maintains stable cellular production rates independent of myocyte metabolic activity (15). Building upon these complementary biochemical profiles, the CRE to CYC ratio (CCR) emerged from Kashani’s research as a practical diagnostic tool for sarcopenia evaluation (16). Subsequent investigations have validated CCR’s prognostic utility across multiple malignancies (17–19).

Nevertheless, the clinical relevance of CCR in EC remains underexplored. To date, CCR may be a useful prognostic indication of surgical complications and long-term survival in patients with EC, as well as an efficient screening tool for sarcopenia (20). However, because of the widespread use in the treatment of EC with NICT, the clinical results and prognosis have drawn more attention in recent years. Additionally, there are currently no reliable and sensitive hematological indices to predict the course of treatment for EC receiving NICT. In this research, we aimed to explore the relationship between pretreatment serum CCR and prognosis in those with ESCC receiving NICT.

## Methods

### Selection criteria

This retrospective cohort research comprised ESCC cases receiving NICT during 2019-2021. Peripheral blood biomarkers and demographic-clinical datasets were systematically collected. Exclusion parameters encompassed: (i) non-squamous pathological subtypes; (ii) concurrent antineoplastic interventions; (iii) incomplete tumor resection post-NICT; (iv) previous or concurrent with other malignancies; (v) coexisting with other hematologic, autoimmune, or inflammatory diseases; and (vi) inadequate clinical records or follow-up durations. **Supplementary Figure S1** delineates the participant selection workflow. Tumor staging in the current research was determined based on the AJCC/UICC TNM staging criteria (8th edition) (21). The institutional review board of Zhejiang Cancer Hospital authorized the research protocol (IRB2020183) in compliance with Helsinki Declaration ethical guidelines.

### Treatment and follow-up

NICT, in the current study, was administered in two cycles at 21-day intervals as part of the preoperative management. The ICIs, such as camrelizumab (200 mg), sintilimab (200 mg), or tislelizumab (200 mg), were included in the regimens on day 1. Chemotherapeutic protocols included albumin-bound paclitaxel (120 mg/m²) on days 1 and 8, combined with carboplatin (AUC=5 mg/mL/min) administered on day 1. Surgical intervention - employing either the Ivor Lewis or McKeown approach - was scheduled for execution between 4–6 weeks post-NICT completion. Current clinical guidelines lack consensus on postoperative adjuvant regimens, though the CheckMate 577 trial suggests adjuvant immunotherapy post-NCRT demonstrates therapeutic advantages (22). Accordingly, selective adjuvant treatment, but not mandatory, was administered post-resection, prioritizing cases with histopathological confirmation of ypN1–3 or ypT3/T4a. Longitudinal monitoring continues through December 2024.

### Statistical analysis

The Kolmogorov-Smirnov test for continuous data in this investigation revealed that all of the data were non-normally distributed. Consequently, the median and interquartile ranges (IQR) were used to present all continuous data, whereas frequency and percentage were also used to report categorical variables. Utilizing the restricted cubic spline (RCS), the ideal threshold for CCR was established based on the examination of the non-linear relationship between disease-free survival (DFS)/overall survival (OS) and CCR. Using measures of variance inflation factor (VIF) and tolerance, the multicollinearity between different independent hematological variables was examined (23). Severe collinearity of the variables was indicated by a tolerance <0.1 or a VIF > 5 (24). The clinical applicability and prognostic evaluation of CCR and other hematological indices were compared and evaluated by using decision curve analyses (DCAs), receiver operator characteristic curves (ROCs), and time-dependent areas under the curves (AUCs). Log-rank tests and Kaplan-Meier analyses were also utilized to compare the DFS/OS between the low and high groups. DFS and OS were assessed using hazards models based on Cox proportional analyses, which were represented as hazard ratios (HRs) with 95% CIs. Firstly, factors with statistical differences were screened out based on univariate regression analysis. Then, a multivariate analysis with stepwise regression was conducted on the above-mentioned statistically significant factors. An established nomogram model was based on the findings of multifactor Cox proportional risk regression analysis. Internal verification was conducted using the bootstrap method, which involves sampling 1000 times. The model’s prediction performance was represented by the concordance index (C-index), and its prediction conformance was visually represented using the calibration approach. DCA assessed the model’s applicability value. The application value of the model was evaluated by ROCs and DCAs. Using R 4.1.2 and SPSS 20.0, all analyses were conducted with a significance criterion of P < 0.05.

## Results

### Patient characteristics

According to inclusion and exclusion criteria, 202 cases were finally eligible to be included in this cohort. There were 20 (9.9%) females and 182 (90.1%) males, with a mean age of 64 years (range: 45–75 years). After surgery, there were 118 (58.4%) patients in the ypT0–2 stage and 84 (41.6%) patients in the ypT3-4a stage. Additionally, 83 patients (41.1%) had positive lymph nodes. Finally, pCR was seen in sixty-one (30.2%) individuals after NICT. After a median follow-up period of 40 months (range: 7–54 months), 77 (38.1%) cases experienced relapses and 54 (26.7%) instances resulted in death.

### Comparisons between hematological indicators and CCR

**Figure 1:**
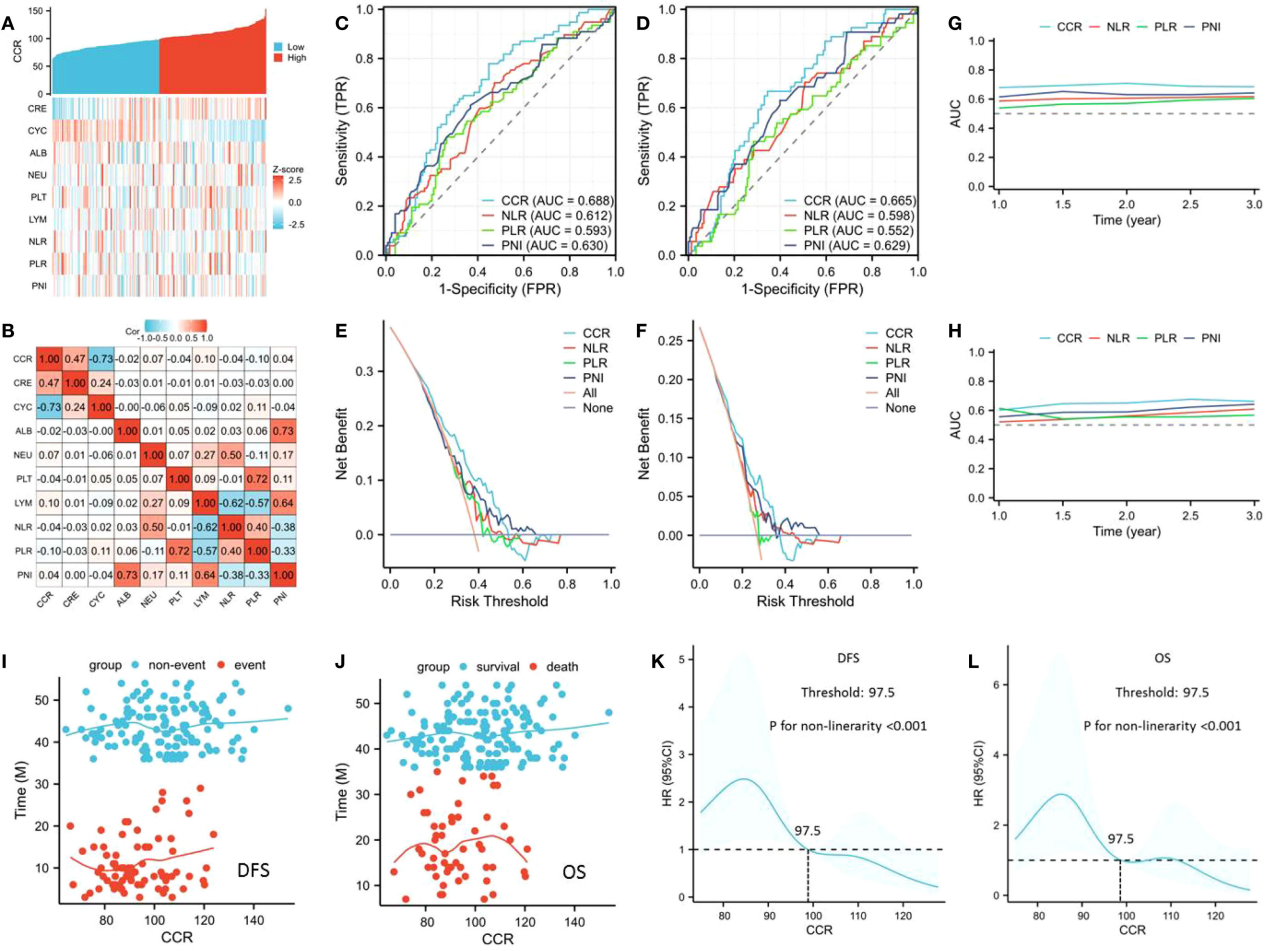
The correlation diagram for all hematological indices **(A, B)**. ROCs for prognostic evaluation in DFS **(C)** and OS **(D)**. DCAs for clinical applicability in DFS **(E)** and OS **(F)**. Time-dependent AUCs for predictive value in DFS **(G)** and OS **(H)**. The connection between CCR and DFS **(I)**/OS **(J)**. The ideal CCR threshold by using an RCS model in DFS **(K)** and OS **(L)**.

In the current study, CCR’s prognostic usefulness was assessed by contrasting it with various traditional hematological parameters including NLR, PLR and PNI. To address the collinearity, VIF and tolerance were included. There was no obvious collinearity between the variables, according to the analysis (VIFs between 1.014 and 1.264 and tolerances between 0.791 and 0.986) (**Supplementary Table S1**). The correlation diagram for all hematological indices is depicted in **Figures 1A, B**. When compared to other hematological indices, the ROCs showed that CCR had the biggest AUC (OS=0.665, DFS=0.688), suggesting a superior capacity for prediction (**Figures 1C, D**). The DCAs also approved of CCR’s greater clinical applicability in both OS and DFS (**Figures 1E, F**). As for the predictive value in the time-dependent AUCs, additionally, CCR again fared better than other traditional indices (**Figures 1G, H**). Therefore, CCR exhibited the strongest clinical application and predictive power among all these indicators.

### Relationships between clinical characteristics and CCR

The link between survival (DFS/OS) and CCR is depicted in **Figures 1I, J**, indicating a non-linear relationship. The optimal CCR cutoff value was determined by the RCS model to be 97.5 (**Figures 1K, L**). A threshold of 97.5 (as the ideal CCR cutoff point) was then used to divide the patients into two groups. There were no statistically significant variations in the other clinical variables between these two cohorts, with the exception of the statistically significant differences in sex (P=0.014), tumor length (P=0.014), and ypT stage (P=0.021) (**Table 1**). Therefore, patients with a low CCR were associated with increased prevalence of ypT disease and tumor length. Regarding postoperative complications, patients with a low CCR were associated with increased prevalence of respiratory complications (P=0.038).

**Table 1:** Patient characteristics grouped by CCR in ESCC receiving NICT.

	Low CCR (97)	High CCR (105)	P-value
Clinical characteristics			
Age (median, IQR, years)	64 (57, 69)	64 (59, 68)	0.942^&^
Sex (female/male, n)	14/83	6/99	0.038^#^
Smoking history (yes/no, n)	64/33	81/24	0.078^#^
Drinking history (yes/no, n)	66/31	77/28	0.409^#^
BMI (median, IQR, Kg/m^2^)	21.6 (20.6, 22.6)	21.8 (20.2, 22.6)	0.956^&^
Tumor location (U/M/L, n) Differentiation (W/M/P, n)	10/55/32 22/43/32	9/62/34 23/47/35	0.898^#^ 0.991^#^
Surgical method (MK/IL, n)	86/11	87/18	0.240^#^
Vessel invasion (yes/no, n)	13/84	11/94	0.521^#^
Perineural invasion (yes/no, n)	17/80	16/89	0.660^#^
Tumor length (median, IQR, cm)	2.4 (0.4, 3.2)	1.5 (0, 2.7)	0.014^&^
pCR (yes/no, n)	24/73	37/68	0.105^#^
ypT stage (T0/T1-2/T3-4a, n)	24/27/46	37/30/38	0.021^#^
ypN stage (N0/N1-3, n)	55/42	64/41	0.539^#^
Adjuvant therapy (yes/no, n)	81/16	79/26	0.148^#^
Intraoperative characteristics			
Operative time (median, IQR, min)	220 (200, 240)	215 (200, 235)	0.280^&^
Operative blood loss (median, IQR, ml)	150 (100, 200)	150 (100, 200)	0.882^&^
Stay after surgery (median, IQR, day)	12 (11, 14)	11 (10, 13)	0.131^&^
Postoperative complications			
Respiratory complications (yes/no, n)	27/70	16/89	0.029^#^
Anastomotic leakage (yes/no, n)	10/87	11/94	0.969^#^
Recurrent nerve injure (yes/no, n)	11/86	12/93	0.984^#^
Chylothorax (yes/no, n)	3/94	1/104	0.558^#^
Hematological indices			
creatinine (median, IQR, μmol/L)	74.2 (67.6, 81.3)	80.3 (75.2, 86.0)	<0.001^&^
cystatin C (median, IQR, mg/L)	0.87 (0.82, 0.93)	0.71 (0.65, 0.76)	<0.001^&^
CCR (median, IQR)	87.1 (79.4, 92.5)	109.1 (104.5, 119.4)	<0.001^&^
albumin (median, IQR, mg/L)	4.09 (3.95, 4.2)	4.12 (3.99, 4.21)	0.407^&^
neutrophil (median, IQR, 10^9^/L)	4.0 (3.5, 4.4)	4.1 (3.6, 4.6)	0.285^&^
platelet (median, IQR, 10^9^/L)	197 (169, 235)	200 (165, 253)	0.605^&^
lymphocyte (median, IQR, 10^9^/L)	1.3 (1.2, 1.6)	1.4 (1.2, 1.6)	0.247^&^
NLR (median, IQR)	2.92 (2.50, 3.31)	2.94 (2.50, 3.28)	0.811^&^
PLR (median, IQR)	145.7 (114.6, 180.0)	144.6 (113.9, 169.3)	0.587^&^
PNI (median, IQR)	47.9 (46.3, 49.3)	48.1 (46.8, 49.7)	0.326^&^

ESCC, esophageal squamous cell carcinoma; NICT, neoadjuvant immunochemotherapy; CCR, creatinine to cystatin C ratio; IQR, interquartile range; U/M/L, upper/middle/lower; BMI, body mass index; W/M/P, well/moderate/poor; MK/IL, McKeown/Ivor Lewis; pCR, pathological complete response; SD, standard deviation; TNM, tumor node metastasis; NLR, neutrophil to lymphocyte ratio; PLR, platelet to lymphocyte ratio; PNI, prognostic nutritional index.

^#^chi-square test; ^&^Mann-Whitney U test.

### Survival grouped by CCR

**Figure 2:**
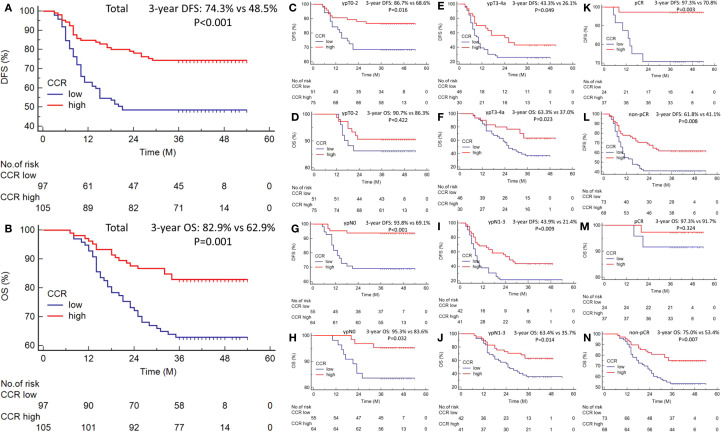
The 3-year DFS [48.5% vs. 74.3%, P<0.001; **(A)**] and OS [62.9% vs. 82.9%, P=0.001; **(B)**] grouped by CCR. Subgroup analysis according to the ypT stage **(C–F)**, ypN stage **(G–J)**, and pCR **(K–N)**. Survival analysis was presented by the Kaplan-Meier curve, and differences were compared by the log-rank test.

Patients exhibiting low CCR demonstrated significantly worse 3-year DFS (48.5% vs. 74.3%, P<0.001; **Figure 2A**) and OS (62.9% vs. 82.9%, P=0.001; **Figure 2B**) compared to those with high CCR. To better understand the prognostic significance of CCR, we conducted a subgroup analysis according to the ypT stage, ypN stage, and pCR. The results of the analyses showed that CCR was able to distinguish the OS and DFS for each subgroup analysis (**Figures 2C–N**). In the subgroup analyses of ypT stage, there were statistically significant differences for DFS (ypT0-2: P=0.016; ypT3-4a: P=0.049), while for OS, it was more statistically significant in the ypT3-4a stage (ypT0-2: P=0.422; ypT3-4a: P=0.023) (**Figures 2C–F**). In the ypN stage subgroup analysis, we found that CCR can effectively stratify the prognosis of ESCC patients in both DFS and OS (**Figures 2G–J**). Similarly, in the pCR subgroup analyses, there were statistically significant differences in DFS, while for OS, there were no statistically significant differences among pCR patients (**Figures 2K–N**). Hence, the results indicated that CCR had prognostic significance for the ESCC cases stratified according to subgroup analyses.

### Predictors of DFS and OS

**Figure 3:**
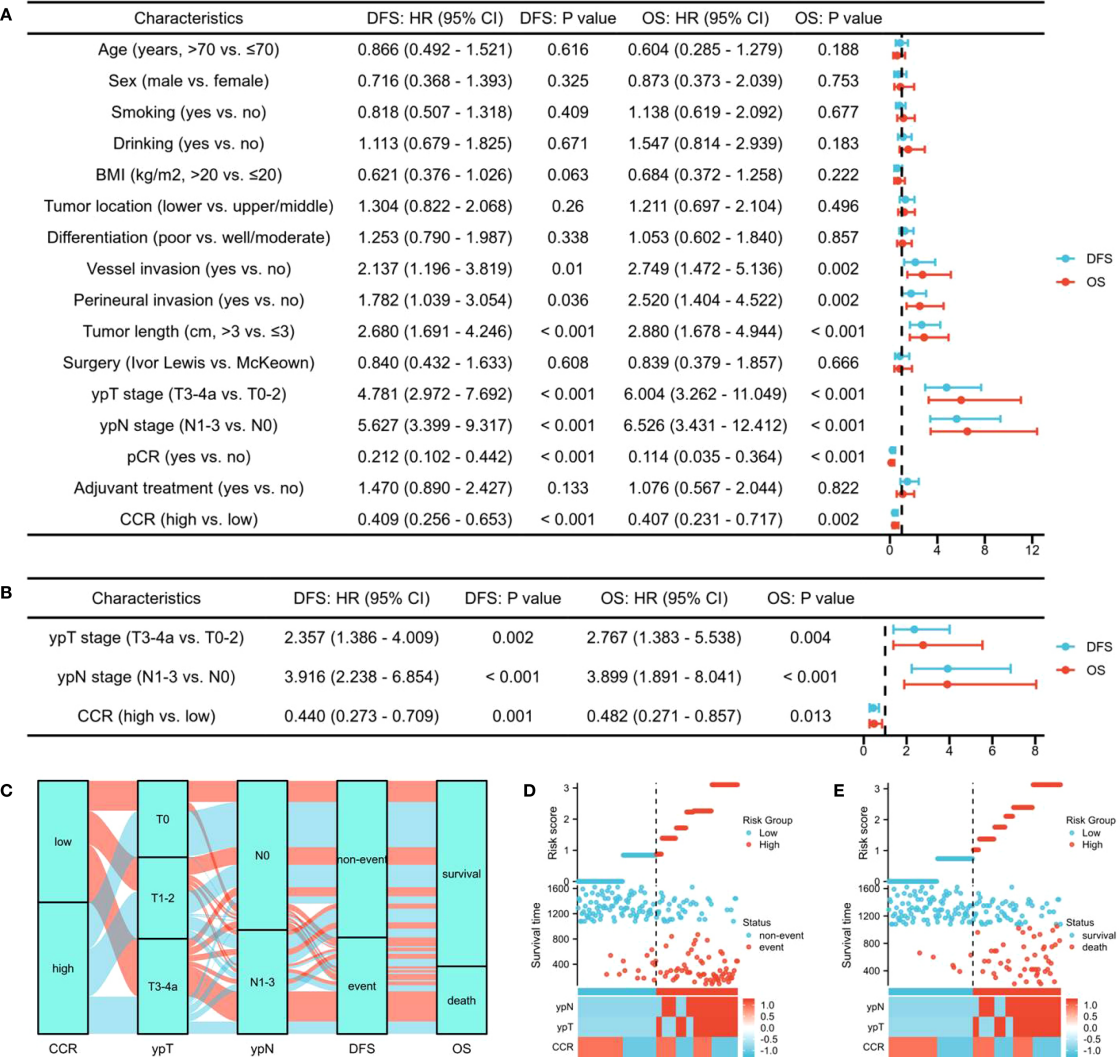
The univariate analysis for DFS and OS **(A)**. Subsequent multivariate analysis for DFS and OS **(B)**. The Sankey diagram regarding tumor stage and prognosis **(C)**. The risk-score analysis in DFS **(D)** and OS **(E)**.

In the current study, RCS showed that with the increase of CCR, the DFS and OS of ESCC patients receiving NICT gradually increased. After correcting for confounding factors, there was still a negative linear relationship between DFS/OS and CCR. In univariate analysis, DFS and OS were significantly affected by the following clinical characteristics: vessel invasion, perineural invasion, tumor length, ypT stage, ypN stage, pCR, and CCR (**Figure 3A**). Subsequent multivariate analysis revealed that CCR was found to be an independent predictor of both OS (HR=0.482, 95% CI=0.271-0.857, P=0.013) and DFS (HR=0.440, 95% CI=0.273-0.709, P=0.001) (**Figure 3B**). Compared to the low CCR group, the high CCR group reduced the risk of recurrence by 56.0% and the risk of death by 51.8%, respectively. The Sankey diagram, which was used to analyze the relationship between clinical outcomes and CCR, revealed that the lowest CCR group was more likely to die and experience recurrence (**Figure 3C**). Finally, we conducted a risk-score analysis based on the three indicators with statistical differences in multivariate regression. Through this analysis, prognosis stratification can be better guided in both DFS (**Figure 3D**) and OS (**Figure 3E**).

### Establishment and verification of nomogram model

Using a nomogram based on statistically significant variables in Cox regression analysis, the model’s prediction outcomes can be visually shown (**Supplementary Figures S2A, B**). The model predicted DFS and OS with a C-index of 0.785 (95% CI: 0.761–0.810) and 0.789 (95% CI: 0.762–0.816), respectively, according to the findings of the model’s prediction ability using the C-index. Internal validation of the model was performed using the Bootstrap self-sampling technique (B=1000). The calibration curve demonstrated that the actual observation probability and the anticipated DFS and OS were reasonably consistent (**Supplementary Figures S2C, D**). To assess the model’s accuracy, ROC curves for 1- and 3-year survival DFS/OS were plotted based on independent parameters (**Supplementary Figures S2E, F**). The model’s application value was assessed using DCA curves, and the findings also demonstrated good clinical applicability (**Supplementary Figures S2G–J**).

## Discussion

Currently, there is a growing interest in the connection between the CCR and the prognosis of cancer patients. According to a study including 3060 patients, CCR was substantially linked to a lower 6-month cancer patient mortality (17). In a different investigation, CCR was found to be independently connected with sarcopenia and relapse-free survival in 413 patients with gastrointestinal stromal tumors who had surgical resection (18). According to another retrospective investigation involving 975 patients with colorectal cancer, CCR may be a useful prognostic index for predicting prognosis, aiding in pathological staging and assisting in in-depth prognostic stratification in conjunction with tumor markers (19). An analysis of 190 individuals with biliary tract cancer revealed that those with a low CCR had a considerably worse prognosis than those with a high CCR (25). According to the current study’s findings, NICT-treated ESCC patients in the high CCR group had a noticeably higher DFS/OS than those in the low group. Multivariate analysis showed that ESCC patients receiving NICT with high CCR reduced the risk of recurrence by 56.0% and the risk of death by 51.8%, respectively.

However, the relationship between ESCC and CCR remains unclear. To date, CCR may be a useful prognostic indication of surgical complications and long-term survival in patients with EC, as well as an efficient screening tool for sarcopenia (20). Nevertheless, there are still significant distinctions between our study and earlier research. First off, while the present study focused on patients with ESCC receiving NICT, the earlier study covered all pathogenic types and stages of EC. This is more in accordance with the current diagnosis and treatment in the context of the immunotherapy era. Second, the information from earlier research was comparatively outdated. The overall treatment approaches and outcomes of EC have greatly improved in comparison to earlier times due to scientific and technological advancements. Last but not least, our study also contrasted CCR with a few conventional hematological indicators, showing that CCR has superior predictive and clinical application. Consequently, CCR enhanced the prognosis of NICT-treated ESCC patients. Therefore, we thought that before starting treatment, CCR might perform an initial evaluation of these patients’ clinical status and prognosis. For patients with low CCR, adjuvant immunotherapy is recommended for those with locally advanced disease, albeit it is not mandatory.

Currently, the specific mechanism between CCR and the prognosis of ESCC patients has not been fully clarified. The CCR’s representation of muscle mass, a known risk factor for cancer, could be one reason for the correlation between the CCR and the outcomes of cancer patients. Numerous studies have shown a significant correlation between the CCR and muscle mass, suggesting that it could be a useful biomarker for muscle in cancer patients (26, 27). Furthermore, the CCR seems to be a reliable marker for sarcopenia diagnosis in cancer patients (28). According to earlier research, patients with high white blood cell counts had low serum CRE levels (29), and those with chronic inflammatory diseases had raised CYC levels (30). As a result, reduced CCR might be linked to a higher burden of inflammation, which has been shown to be a poor prognostic marker for cancer patients (31). Increased inflammatory burden is a significant factor influencing the prognosis of cancer patients, and systemic inflammation is the most characteristic interaction between tumor and host (32). Therefore, the CCR may be a promising prognostic indicator.

It is important to recognize that the existing research has several limitations. Firstly, the sample size is small given the limited amount of CCR available in our patients and the recent acquisition of these data. In the current retrospective study, we confirmed that CCR can serve as an important indicator of neoadjuvant immunotherapy and is of great significance for prognosis, thereby demonstrating certain clinical significance. Secondly, the explanation of the relationship between CCR and muscle mass is further limited by the absence of data to assess sarcopenia in this study. However, compared to sarcopenia, which is costly, time-consuming, and requires specialist software and knowledge, the straightforward assessment method of CCR makes it an appealing candidate for use as a prognostic factor. Thirdly, even though this study had stringent inclusion and exclusion criteria, CCR, a novel hematological parameter, could be impacted by other variables, which would change the findings. Fourthly, the discriminative capacity of CCR falls below the conventional threshold for “good” prediction. However, it may be rare to achieve a good predictive effect merely through hematological indicators. At present, PD-L1 and minimal residual disease (MRD) may be considered significant in the immune era, but these biomarkers are currently relatively expensive and the detection process is relatively complicated (33, 34). Therefore, it is highly necessary in clinical practice to predict the prognosis through some simple blood indicators. Although the CCR may not have reached a very high quality, it still holds certain significance for clinical practice and is also helpful for subsequent clinicians to explore joint predictions to achieve better predictive effects. Fifthly, we were unable to investigate the effect of CCR trajectory alterations on prognosis because this study only gathered serum CCR data once. CCR was measured only at pretreatment, limiting insights into how dynamic changes in CCR might affect outcomes. Therefore, future studies could evaluate serial CCR measurements to assess its trajectory as a prognostic index. Finally, another weakness of this study is the absence of an independent validation cohort. We also hope that more researchers can be inspired by our research and thus conduct more extensive multi-center prospective clinical studies to confirm the corresponding results. Therefore, longer-term prospective validation is still required to have a better understanding of CCR’s capacity to predict long-term prognosis.

## Conclusions

In summary, the therapeutic efficacy of NICT for ESCC can be predicted by pretreatment CCR. Although the CCR may not have attained a very high quality, it still holds certain significance for clinical practice in ESCC patients undergoing NICT.

## Data Availability

The original contributions presented in the study are included in the article/**Supplementary Material**. Further inquiries can be directed to the corresponding author/s.

## Supplementary material

Supplementary Table 1 Multicollinearity analysis of various hematological indices. CCR: creatinine to cystatin C ratio; NLR: neutrophil to lymphocyte ratio; PLR: platelet to lymphocyte ratio; PNI: prognostic nutritional index; VIF: variance inflation factor.

Supplementary Figure 1 The participant selection workflow.

Supplementary Figure 2 Nomogram models in DFS **(A)** and OS **(B)**. Calibration curves for 1-and 3-year DFS **(C)** and OS **(D)**. ROC curves for 1- and 3-year DFS **(E)** and OS **(F)**. DCA curves demonstrated good clinical applicability of 1- and 3-year DFS **(G, H)** and OS **(I, J)**.
