# Supplementary figures and images for: Serum creatinine to cystatin C ratio is a prognostic indicator in esophageal squamous cell carcinoma receiving neoadjuvant immunochemotherapy
Source: Front Immunol. 2025 Sep 17;16:1645874. doi: 10.3389/fimmu.2025.1645874 (PMC12484131; doi:10.3389/fimmu.2025.1645874)

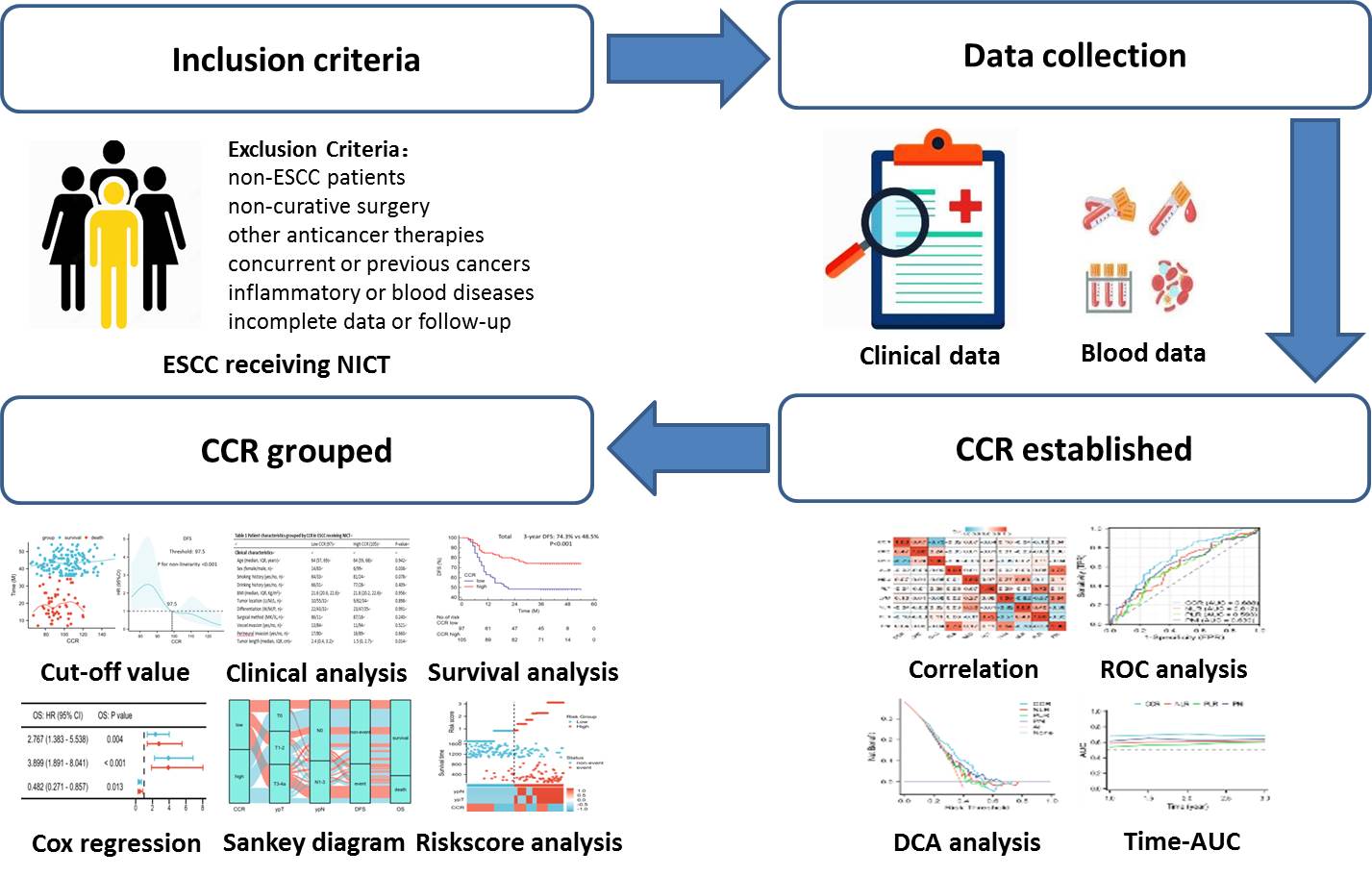

Supplement: Supplementary Figure 1 — The participant selection workflow. [file Image1.jpeg]

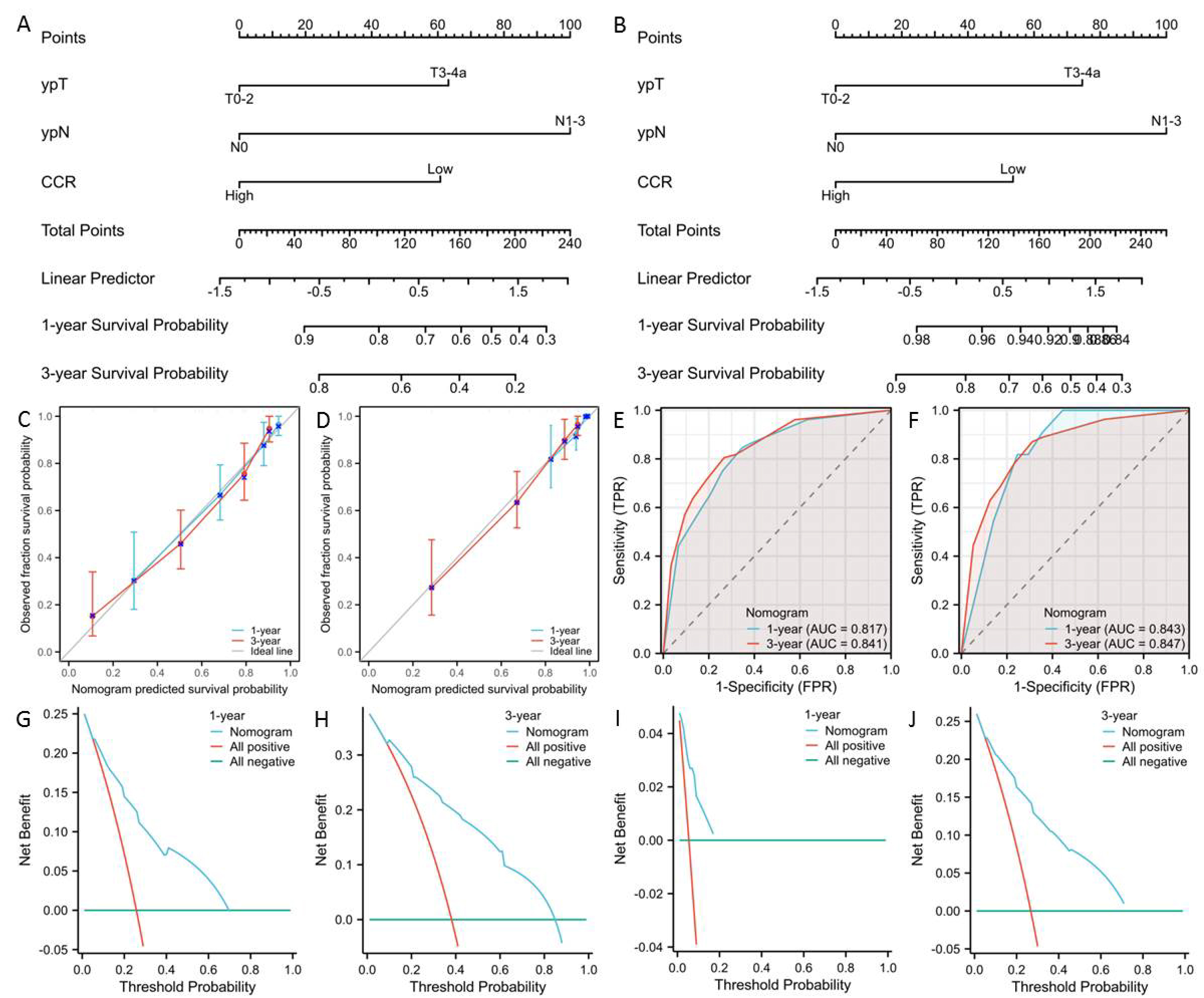

Supplement: Supplementary Figure 2 — Nomogram models in DFS (A) and OS (B). Calibration curves for 1-and 3-year DFS (C) and OS (D). ROC curves for 1- and 3-year DFS (E) and OS (F). DCA curves demonstrated good clinical applicability of 1- and 3-year DFS (G, H) and OS (I, J). [file Image2.jpeg]
