# Supplementary material for: Serum creatinine to cystatin C ratio is a prognostic indicator in esophageal squamous cell carcinoma receiving neoadjuvant immunochemotherapy
Source: Front Immunol. 2025 Sep 17;16:1645874. doi: 10.3389/fimmu.2025.1645874 (PMC12484131; doi:10.3389/fimmu.2025.1645874)
Supplement: Supplementary file 4 [file Table2.docx]

**Table S1 Multicollinearity analysis of various hematological indices**

|  | Tolerance | VIF |
| --- | --- | --- |
| NLR  PLR  PNI  CCR | 0.791  0.793  0.846  0.986 | 1.264  1.260  1.181  1.014 |

**Abbreviation:** CCR: creatinine to cystatin C ratio; NLR: neutrophil to lymphocyte ratio; PLR: platelet to lymphocyte ratio; PNI: prognostic nutritional index; VIF: variance inflation factor.
